# Neural sensitivity to social reward and punishment anticipation in social anxiety disorder

**DOI:** 10.3389/fnbeh.2014.00439

**Published:** 2015-01-05

**Authors:** Henk R. Cremers, Ilya M. Veer, Philip Spinhoven, Serge A. R. B. Rombouts, Karin Roelofs

**Affiliations:** ^1^Radboud University Nijmegen, Behavioral Science InstituteNijmegen, Netherlands; ^2^Biological Science Department, Department of Psychiatry, University of ChicagoChicago, IL, USA; ^3^Leiden Institute for Brain and CognitionLeiden, Netherlands; ^4^Department of Radiology, Leiden University Medical CenterLeiden, Netherlands; ^5^Division of Mind and Brain Research, Department of Psychiatry and Psychotherapy, Charité UniversitätsmedizinBerlin, Germany; ^6^Institute of Psychology, Leiden UniversityLeiden, Netherlands; ^7^Department of Psychiatry, Leiden University Medical CenterLeiden, Netherlands; ^8^Donders Institute for Brain, Cognition and BehaviorNijmegen, Netherlands

**Keywords:** social anxiety disorder, fMRI, reward, punishment, social incentives

## Abstract

An imbalance in the neural motivational system may underlie Social Anxiety Disorder (SAD). This study examines *social* reward and punishment anticipation in SAD, predicting a valence-specific effect: increased striatal activity for punishment avoidance compared to obtaining a reward. Individuals with SAD (*n* = 20) and age, gender, and education case-matched controls (*n* = 20) participated in a functional magnetic resonance imaging (fMRI) study. During fMRI scanning, participants performed a Social Incentive Delay (SID) task to measure the anticipation of social reward and punishment. The left putamen (part of the striatum) showed a valence-specific interaction with *group* after correcting for medication use and comorbidity. The control group showed a relatively stronger activation for reward vs. punishment trials, compared to the social anxiety group. However, *post-hoc* pairwise comparisons were not significant, indicating that the effect is driven by a relative difference. A connectivity analysis (Psychophysiological interaction) further revealed a general salience effect: SAD patients showed decreased putamen-ACC connectivity compared to controls for both reward and punishment trials. Together these results suggest that the usual motivational preference for social reward is absent in SAD. In addition, cortical control processes during social incentive anticipation may be disrupted in SAD. These results provide initial evidence for altered striatal involvement in both valence-specific and valence-nonspecific processing of social incentives, and stress the relevance of taking motivational processes into account when studying social anxiety.

## Introduction

Avoidance motivation is a core aspect of social anxiety disorder (SAD; Neal and Edelmann, 2003; Holtforth, 2008). Research over the last decades has identified a dopaminergic-mediated brain circuit involved in motivational processing (Haber and Knutson, 2010). This system centers around the ventral parts of the striatum, and is connected to several other regions, including prefrontal control regions such as the orbitofrontal cortex and the anterior cingulate cortex (ACC; Haber and Knutson, 2010). Anticipatory striatal activity is thought to reflect *motivational salience*, and is linked to both appetitive (reward) and aversive (punishment avoidance) motivation (Salamone, 1994). However, SAD may be associated with an imbalance in the striatal motivational system (valence-specific effect), possibly due to a relatively stronger motivational drive to avoid social punishments.

A recent model integrates Reinforcement Sensitivity Theory (a model for reward, punishment, and motivation processing) and SAD, and highlights the role of behavioral inhibition as a temperamental predisposition to the development of social anxiety (Kimbrel, 2008). The behavioral inhibition system is linked to punishment or threat sensitivity as well as to the motivation to avoid potentially harmful situations (i.e., harm avoidance Carver and White, 1994). Based on this theory, one may expect that the striatal motivational system shows a differential preference for reward sensitivity and punishment avoidance, either reflecting the absence of a motivational drive to obtain a reward, a heightened motivation to avoid punishments, or both.

Brain imaging research on SAD has identified several regions showing differential patterns of activity and structure compared to controls, including the ACC, amygdala, the insula, and medial prefrontal cortex (Etkin and Wager, 2007; Freitas-Ferrari et al., 2010; Fouche et al., 2013; Hattingh, 2013; Brühl et al., 2014). In addition, several lines of research have indeed linked SAD to alteration of striatal activity and dopamine levels, see Freitas-Ferrari et al. (2010) for an overview. Much remains unknown however, on the direction (increased or decreased) and valence-specificity (reward or punishment) of these shifts in the motivational brain systems.

Important evidence comes from work highlighting the role of the striatal motivational systems in behaviorally inhibited adolescents, who are at risk for developing SAD (Helfinstein et al., 2012). fMRI studies in adolescents with a history of behavioral inhibition (Guyer et al., 2006) and adolescents with SAD (Levita et al., 2012) found overall increased activation in the ventral striatum, not only for impending monetary rewards, but also for punishments. This valence-nonspecific increase in striatal activity was interpreted as reflecting a general motivation to avoid making mistakes. Interestingly, a study comparing social and non-social rewards in SAD found that social anxiety was related to a stronger striatal activation for monetary rewards, but weaker activation for social rewards (Richey et al., 2014), perhaps reflecting a dissociation between these two types of incentives.

Here we investigated brain activation and connectivity in SAD during the anticipation of obtaining a social reward and avoiding a social punishment. We compared both valence-specific effects (different between reward and punishment) and general motivational salience effects (similar for reward and punishment). Based on the theory that SAD patients have a stronger motivation to avoid harm, we hypothesized that this group would show greater striatal activation when avoiding social punishments than when obtaining social rewards. In addition, we investigated striatal connectivity with regions that are part of the motivation network, such as the ACC, hypothesizing an increased need for regulatory control in SAD during social incentive processing.

## Methods

### Participants

This study included 20 participants with SAD and 20 healthy control participants (HC) selected from a pool of 24 subjects matching in age, gender, and years of education (see Table 1). Participants completed several questionnaires: Liebowitz Social Anxiety Scale (LSAS; Fresco et al., 2001), Social Phobia Anxiety Inventory (SPAI; Turner et al., 1989), Brief Fear of Negative Evaluation (BFNE; Weeks et al., 2005), Beck Depression Inventory (BDI; Beck et al., 1988), the five-factor model of personality (NEO-FFI; Costa and McCrea, 1992), and the Behavioral Activation and Behavioral Inhibition Scale (BIS/BAS; Carver and White, 1994), see Table 1. SAD participants were recruited through local participating treatment centers (*n* = 8), advertisement (*n* = 7), and social anxiety websites (*n* = 5). Inclusion criteria for social anxiety participants were an LSAS score of 60 or higher, and meeting criteria for general SAD (as a primary diagnosis) according to the DSM-IV (1994) as assessed by the Mini-International Neuropsychiatric Interview (MINI). The MINI is a well- validated diagnostic instrument (Sheehan et al., 1997) and took approximately 45 min to complete for SAD participants. Two SAD participants had a secondary comorbid depressive episode, while four others had a history of depressive episodes. Two of these SAD participants were on stable Selective Serotonin Reuptake Inhibitor (SSRI) use. Healthy control participants had no history of psychiatric disease or psychotropic medication use. The study was approved by the Medical Ethical Committee of the Leiden University Medical Center, and written informed consent was given by all participants.

**Table 1:** **Participant characteristics**.

**Mean (SD)**				
	**Social anxiety**	**Control subjects**	***F*-value**	***p*-value**
	**(*n* = 20)**	**(*n* = 20)**		
Age, year	29.1 (7.5)	27.7 (7.7)	0.33	0.57
Gender, male/female	11/9	11/9		
Years of education	16 (2.4)	16.4 (2.2)	0.26	0.61
LSAS	85.9 (13.9)	21.6 (13.1)	225.23	<0.001
BDI	20.5 (11.6)	5.2 (4,4)	40.52	<0.001
SPAI-SP	136.3 (21.3)	49.8 (24.9)	132.9	<0.001
BFNE	54.3 (5.6)	36.0 (9.2)	44.59	<0.001
NEO-N	43.6 (9.8)	29.5 (6.7)	24.54	<0.001
NEO-E	30.8 (6.3)	42.7 (4.8)	39.51	<0.001
BIS	24.7 (3.4)	18.5 (4.2)	25.7	<0.001
BAS-Reward	14.9 (2.3)	16.6 (2.2)	5.8	0.021

*LSAS, Liebowitz social anxiety scale; SPAI-SP, Social Phobia Anxiety Inventory – Social Phobia subscale; BFNE, brief fear of negative evaluation; BDI, Beck depression inventory; NEO-N, NEO-FFI neuroticism; NEO-E, NEO-FFI Extraversion; BIS, Behavioral Inhibition System; BAS, Behavioral Activation System*.

### Materials and procedures

**Figure 1:**
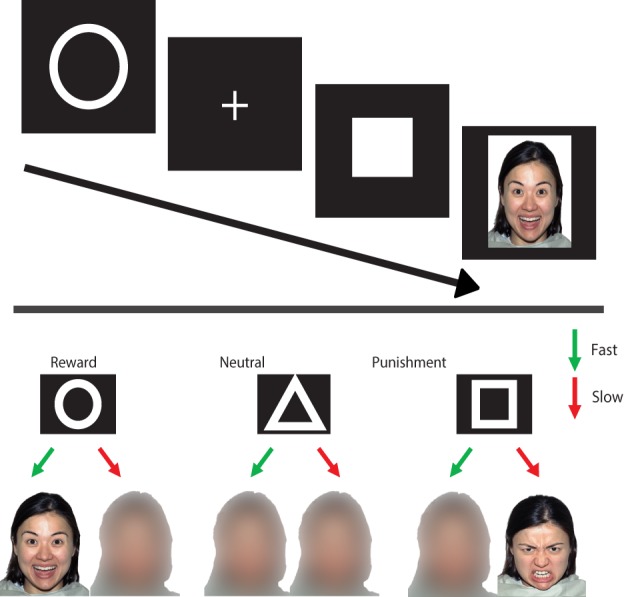
**Social incentive delay task. Upper panel:** on each trial, a cue of (500 ms) (indicating the condition) is followed by a delay period (1500–3500 ms) after which a target is presented (150–500 ms). When the target is shown, participants are instructed to press a button as fast as possible. Depending on whether the reaction time is fast enough, one of two possible feedback screens appear (1650 ms). **Lower panel:** the different conditions with the associated feedback (outcome).

Participants performed the Social Incentive Delay task (SID; Spreckelmeyer et al., 2009), which is a variation of the Monetary Incentive Delay task (MID; Knutson et al., 2001), designed to measure brain activity related to social rewards. In addition, we added a social punishment condition in order to directly compare the punishment and reward conditions. Participants were cued at the start of each trial on the possible outcome when a target detection response (pressing a button with right hand index finger) fell within the presentation time of that target. In the social reward condition, happy faces were the outcome of a fast response (hit) and morphed faces that of a slow response (miss). In the social punishment condition, the morphed faces represented a hit, while angry faces represented a miss. In the control condition, a morphed face was always the outcome, regardless of whether the response was fast enough (see Figure 1).

The task consisted of two runs of 72 trials each. Each trial started with a 500 ms cue, a circle for the reward condition (*n* = 27), a triangle for the neutral condition (*n* = 18), and a square for the punishment condition (*n* = 27). A fixation cross was then presented (for 2250–2750 ms). The combination of the two signs was referred to as the *anticipation period*. The target (filled white square) was presented, and participants were instructed to respond as fast as possible when the target appeared. To ensure that the hit rate in the different conditions was similar across participants, the target duration was variable (160–500 ms) and shortened with 10 ms for the subsequent trial when the previous target was met. The target duration was increased with 20 ms in the subsequent trial when the previous target was missed. This algorithm lead to an approximate hit rate of 66%. The target was followed by the *outcome* (1650 ms), after which a black screen was presented (2500–5000 ms). The different trial types were presented intermixed in an event-related design, with the presentation order of trial types optimized using a genetic algorithm toolbox (Wager and Nichols, 2003).

The faces used in this task were taken from a standardized and validated set of facial expressions, the NIMSTIM database (Tottenham et al., 2009). Both happy and angry expressions of 9 male and 9 female models were used. The morphed faces were generated using a Gaussian smoothing kernel in Adobe Photoshop (www.adobe.com/Photoshop).

Before the actual task, participants completed practice trials until 10 hits were obtained (irrespective of condition). After the scan protocol, participants rated how much each cue was liked on a 11-point Likert scale ranging from 0 (extremely disliked) to 10 (extremely liked), 5 being neutral.

The adaptive reinforcement schedule resulted in the following hit rates: in the control group, the observed mean percentages of hits (±standard error) were 61.0% (±0.014%) for reward, 61.0% (±0.014%) for punishment, and 57% (±0.019%) for the neutral conditions. In the social anxiety group, these were 61.0% (±0.012%) for reward, 61.0% (±0.011%) for punishment, and 54% (±0.02%) for the neutral conditions.

### fMRI data acquisition and preprocessing

Imaging data were acquired on a Philips 3.0-T Achieva MRI scanner using an eight-channel SENSE head coil for radiofrequency transmission and reception (Philips Medical Systems, Best, The Netherlands). Whole-brain fMRI data were acquired using T2^*^-weighted gradient echo echo-planar imaging (EPI) with the following scan parameters: 298 volumes; 38 axial slices scanned in ascending order; repetition time (TR) = 2200 ms; echo time (TE) = 30 ms; flip angle = 80°; FOV = 220 × 220 mm; 2.75 mm isotropic voxels with a 0.25 mm slice gap. A high-resolution anatomical image (T1-weighted ultra-fast gradient-echo acquisition; TR = 9.75 ms; TE = 4.59 ms; flip angle = 8°; 140 axial slices; FOV = 224 × 224 mm; in-plane resolution 0.875 × 0.875 mm; slice thickness = 1.2 mm), and a high-resolution T2^*^-weighted gradient echo EPI scan (TR = 2.2 s; TE = 30 ms; flip angle = 80°; 84 axial slices; FOV = 220 × 220 mm; in-plane resolution 1.96 × 1.96 mm, slice thickness = 2 mm) were acquired for registration to standard space. Data were analyzed using FSL Version 4.1.3 (FMRIB's Software Library, www.fmrib.ox.ac.uk/fsl).

The following preprocessing steps were applied to the EPI data sets: removal of non-brain tissue, spatial smoothing using a Gaussian kernel of 6 mm full width at half maximum (FWHM), grand-mean intensity normalization of the entire 4D dataset by a single multiplicative factor, and a high pass temporal filter of 60 s (i.e., ≥0.016 Hz). The dataset was registered to the high-resolution EPI image, the high-resolution EPI image to the T1-weighted image, and the T1-weighted image to the 2 mm isotropic MNI-152 standard space image (T1-weighted standard brain averaged over 152 subjects; Montreal Neurological Institute, Montreal, QC, Canada). The resulting transformation matrices were then combined to obtain a native to MNI space transformation matrix and its inverse (MNI to native space).

### Analysis

#### Behavioral data

Behavioral data were analyzed with PASW Statistics, Release Version 18 (SPSS, Inc., Chicago, IL, www.spss.com) using a Group (SAD and controls) x Condition (social reward, punishment and neutral) repeated measures ANOVA on mean target reaction times. With respect to the group × condition interaction, comparing two opposing models (contrasts) was of main interest. The first contrast compared reward directly to punishment trials, referred to as the *valence*-*specific effect*. The second planned comparison combined reward and punishment and contrasted these to neutral trials, referred to as the *general salience effect* (valence unspecific). The same analysis was performed for the subjective ratings of the cues. The same model was subsequently run with medication use (SSRI) and comorbidity status as additional covariates. *Post-hoc t*-tests were performed on the pairwise comparisons of conditions, both between and within groups, and reported when *p* < 0.05. Additionally it will be indicated when the *post-hoc* tests also survived bonferoni-correction for multiple comparisons (*p* < 0.0055; this will be abbreviated as bc). Lastly, Spearman correlational analyses were performed to test for a relation between behavioral variables (reaction time data, subjective ratings, social anxiety symptoms; LSAS, and motivational drives; BIS/BAS) and both brain activity and connectivity in the social anxiety group, applying a bonfereroni-correction for multiple comparisons.

#### fMRI data

Time-series statistical analysis was carried out using FILM (FMRIB's Improved Linear Model) with local autocorrelation correction (Smith et al., 2004). Explanatory variables (EVs) were included in the general linear model that modeled the anticipation of reward, anticipation of punishment, and control condition. For the outcome phase, four separate EVs were entered for hits and misses in the reward and punishment conditions, and one EV for the outcome in the neutral condition. One EV was further added to model trials where no response was given at all, while another EV was modeled for all the button presses during the target presentation to explain variance due to motor responses. Each EV was convolved with a double gamma hemodynamic response function to account for the hemodynamic delay, and in addition, the temporal derivative for each EV was included. Contrasts were generated that compared each anticipation condition against the “implicit baseline”: reward > baseline, neutral > baseline, punishment > baseline and against each other: reward > neutral, punishment > neutral, reward+punishment > neutral (general salience contrast) reward > punishment (valence-specific contrast).

In order to increase statistical power, further statistical analyses were restricted to a limited set of brain regions related to reward and punishment based on meta-analytic data and the main effect of task condition across participants. First, the automated meta-analytic database Neurosynth (Yarkoni et al., 2011) was used to create reverse inference statistical maps related to the terms “reward” and “punishment,” which were subsequently combined into one map (see Supplementary Figure 1). This statistical map was used as a region of interest in a voxel-wise analysis of the two main effects of task (reward > baseline and punishment > baseline), cluster-corrected with an initial cluster-forming threshold of *z* > 2.3, and a corrected *p* < 0.05 (see the Supplementary Material for the voxel-wise analysis and results). The conjunction of the two cluster-corrected maps (voxels showing significant activation in both the reward and the punishment conditions) was thus a result of regions known to be involved in reward and/or punishment processing a priori, and those which showed sensitivity to this version of the SID task. Subsequently, to test for a Group × Condition interaction, mean parameter estimates (beta values) were extracted for each region and condition (against the implicit baseline). Subsequently, these were entered per region in a repeated measure ANOVA with Group (SAD and Controls) as between, and Condition (Reward, Punishment, and Neutral) as within-subjects factors. Gray matter density values within the region tested were entered as covariates. The same model was then run with medication use (SSRI) and comorbidity status as additional covariates. Similar to the behavioral data analysis, for the group × condition interaction two-planned comparisons (contrasts) on Condition were performed. The first comparison contrasted reward to punishment trials (valence-specific effect). The second planned comparison combined reward and punishment contrasted to neutral trials (general salience effect).

#### Functional connectivity analyses

In order to examine functional connectivity with the main left putamen cluster identified in the previous analysis (see Results section), a Psychophysiological Interaction (PPI) analysis was performed. This type of analysis can reveal regions that show a task-related change in connectivity with a seed region. Interaction between each of the EVs of interest (Reward, Neutral and Punishment anticipation) and the mean time series of the left putamen was added to the model of each subject, as well as the time series itself. Contrasts were generated testing the effect of each interaction EV (PPI effect) as well as the difference between those effects. The contrast estimates were subsequently analyzed in a group-level analysis.

A voxel-wise analysis of the two main effects of Task (reward > baseline and punishment > baseline, including the reverse contrasts) was performed applying a cluster-correction with an initial whole brain cluster-forming threshold of *z* > 2.3, and a corrected *p* < 0.05. Significant clusters for both the reward and punishment contrasts were further restricted to regions within a meta-analytic connectivity map of the left putamen cluster generated using Neurosynth (Yarkoni et al., 2011), and within regions of the “reward circuit“ (Haber and Knutson, 2010). This procedure resulted in a cluster comprising the ACC and supplementary motor areas (peak coordinate *x* = −6, *y* = 8, *z* = 40), which showed overall task-related changes in connectivity with the putamen seed (see Supplementary Figure 2). Contrast estimates for this cluster were extracted, and tested for potential Group × Condition interactions with a contrast procedure identical to the one used in the behavioral and neural activation analyses.

## Results

### Behavioral results

#### Reaction time (RT)

Mean RTs and standard errors per group and condition are presented in Table 2. A repeated measures ANOVA with Group as a between-subjects and Condition as within-subjects factor showed a main effect of Condition on RT data [*F*_(2, 76)_ = 7.35, *p* = 0.001], and a significant effect on the general salience (valence-unspecific) contrast [*F*_(1, 38)_ = 11.19, *p* = 0.002]. Overall, reaction times on the reward and punishment trials were lower than on the neutral trials. For the Group × Condition interaction, the valence-specific contrast did not show a significant effect (*p* = 0.509) but the general salience contrast did [*F*_(1, 38)_ = 4.27, *p* = 0.045]. Adding SSRI use and comorbidity as a covariate, showed a similar pattern: the valence-specific contrast interaction with Group was non-significant [*F*_(1, 36)_ = 1.17, *p* = 0.286] but the general salience effects was significant [*F*_(1, 36)_ = 5.75, *p* = 0.022]. Reaction times on neutral trials compared to reward and punishment ones were slower in the social anxiety group than in the control group. *Post-hoc* tests revealed that the groups differed significantly on punishment vs. neutral trials [*t*_(38)_ = 2.08, *p* = 0.044], and within the social anxiety group, reaction times were significantly slower for neutral compared to reward [*t*_(19)_ = 2.76, *p* = 0.013] and neutral compared to punishment trials [*t*_(19)_ = 3.12, *p* = 0.006].

**Table 2:** **Behavioral data**.

**Mean (SE)**				
	**Reaction time (ms)**		**Subjective rating**	
	**Social anxiety**	**Control subjects**	**Social anxiety**	**Control subjects**
	**(*n* = 20)**	**(*n* = 20)**	**(*n* = 20)**	**(*n* = 20)**
Reward	240.3 (6.5)	236.0 (6.5)	7.1 (0.4)	7.1 (0.3)
Neutral	253.6 (6.1)	239.9 (6.9)	5.4 (0.4)	4.1 (0.3)
Punishment	238.4 (6.7)	237.0 (6.7)	4.8 (0.4)	6.0 (0.4)

*Reaction times are in milliseconds. Subjective ratings are based on an 11-point likert scale on how much each cue, i.e., the start of a trial, was liked (0, extremely disliked; 10, extremely liked)*.

#### Subjective ratings of cues

Mean subjective ratings and standard errors are presented in Table 2. A mixed ANOVA for the subjective (like-dislike) ratings of the symbols, signaling the condition at the start of each trial, yielded a main effect of Condition: both the valence-specific (*p* < 0.001) and general salience (*p* < 0.001) contrast were significant, indicating that reward trials were liked better than punishment trials, and both reward and punishment trials were liked better than neutral trials. With respect to the Group × Condition interaction, the valence-specific contrast showed a trend [*F*_(1, 38)_ = 3.55, *p* = 0.067] and the general salience contrast showed a significant effect [*F*_(1, 38)_ = 12.91, *p* = 0.001]. Adding SSRI use and comorbidity status as a covariate showed a similar pattern: a trend significant interaction with the valence-specific contrast [*F*_(1, 36)_ = 3.34, *p* = 0.076], and a significant general salience interaction effect [*F*_(1, 36)_ = 10.22, *p* = 0.003]. Overall, social anxious rated the neutral trials lower (compared to reward and punishment) than the control group and showed a trend for disliking the punishment trials more than the reward trials, compared to controls. *Post-hoc* tests revealed that the groups differed on both reward vs. neutral [*t*_(38)_ = 2.57, *p* = 0.014] and punishment vs. neutral [*t*_(38)_ = 3.47, *p* = 0.001]. Within the control group all pairwise comparisons were significant (all *p* < 0.02), and within the social anxiety group reward vs. neutral [*t*_(19)_ = 5.4, *p* < 0.001, bc] as well as reward vs. punishment were significant [*t*_(191)_ = 5.1, *p* < 0.001, bc].

### fMRI results

#### Activation

**Figure 2:**
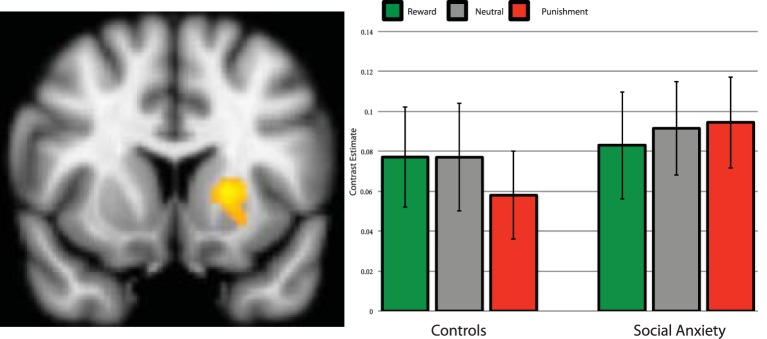
**Brain activation during anticipation in the left putamen region of interest**. Parameter estimates per group and condition compared to baseline. Left side of the image is right side of the brain. Error bars are standard error of the mean.

The analytic procedure identified two clusters that showed a main effect of Task (reward > baseline, and punishment > baseline) within reward and punishment related regions, one in the Putamen (*x* = −20, *y* = 12, *z* = 4, *k* = 242) and another in the thalamus (*x* = 4, *y* = −24, *z* = 6, *k* = 409). The Group × Condition interaction showed a trend significant valence-specific contrast [*F*_(1, 37)_ = 4.057, *p* = 0.051], but did not show the general salience effects (*p* = 0.72), see Figure 2. Adding SSRI use and comorbidity status as a covariate, showed a significant Group × Condition interaction for the valence-specific contrast [*F*_(1, 35)_ = 4.89, *p* = 0.034], but not for the general salience effects (*p* = 0.64). Controls appear to show relatively stronger activation for reward than punishment trials, whereas SAD participants do not demonstrate this differentiation. The *post-hoc* test showed that within the control group, reward vs. punishment was trend significant [*t*_(19)_ = 2.05, *p* = 0.055]. The cluster in the thalamus showed no significant Group × Condition interaction, neither for the valence-specific nor for the unspecific contrast (all *p* > 0.15).

#### Connectivity

**Figure 3:**
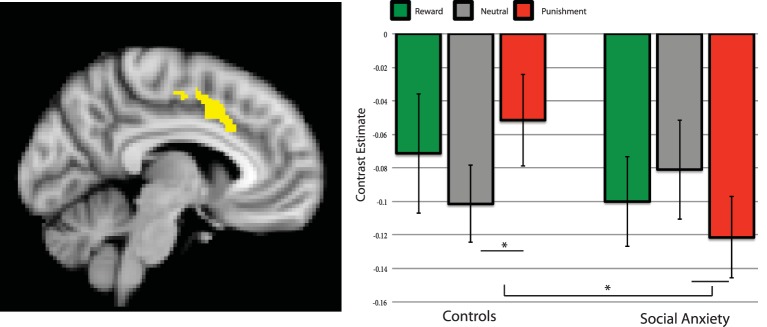
**Brain connectivity between left putamen and dACC during the anticipation of reward, neutral and punishment trials**. Parameter estimates per group and condition compared to baseline. Left side of the image is right side of the brain. Error bars are standard error of the mean. ^*^significant *post-hoc* effect, *p* < 0.05.

Similar to the activation effects, the cluster of the PPI procedure was analyzed using a repeated measures ANOVA, testing for Group × Condition interactions and comparing the planned contrast valence-specific and unspecific (general salience) effects. The valence-specific contrast showed no significant interaction with group [*F*_(1, 37)_ = 1.92, *p* = 0.17], but the general salience contrast did [*F*_(1, 37)_ = 5.33, *p* = 0.027], see Figure 3. Adding SSRI use and comorbidity status as a covariate, showed a similar pattern of significance; the Group × Condition interaction for the valence-specific contrast was non-significant [*F*_(1, 35)_ = 2.02, *p* = 0.164], the general salience effects was significant [*F*_(1, 35)_ = 8.39, *p* = 0.006]. The controls showed less negative Putamen-ACC connectivity for the reward and punishment trials compared to neutral trials, whereas the social anxiety group showed stronger negative Putamen-ACC connectivity for reward and punishment compared trials to neutral ones. *Post-hoc* tests revealed that the groups differed significantly on punishment vs. neutral [*t*_(38)_ = 3.06, *p* = 0.004, bc], and within the control group the punishment vs. neutral comparison was also significant [*t*_(19)_ = 2.49, *p* = 0.02] see Figure 3.

#### Correlation between neural and behavioral data

No significant multiple comparisons corrected correlations between any behavioral measure (symptom severity, reaction time, subjective ratings) and neural measure (activation, connectivity) were found in the social anxiety group.

## Discussion

In this study, we tested whether participants with SAD showed differential neural activity during the anticipation of social reward or punishment. A cluster in the left putamen was identified that showed sensitivity to this task as well as involvement in reward and punishment processing. Within this region, evidence was found for a valence-specific effect. Controls demonstrated relatively stronger activity during the anticipation of reward vs. punishment trials compared to SAD patients. These findings suggest that the relative motivational preference for social reward trials observed in controls is absent in SAD patients, who perhaps have a preference to avoid a social punishment. Note however, that the interaction was only significant when controlling for the use of medication and comorbidity; without taking this into account the effect was trend significant (*p* = 0.051). In addition, the fact that none of the *post-hoc* tests were significant, underscores that this is a relative effect. Connectivity results showed that the social anxiety group demonstrated stronger negative connectivity with the ACC irrespective of reward or punishment conditions. The ACC is known to be involved in a variety of cognitive control functions, such as conflict monitoring (Botvinick et al., 2004), and is also relevant for comparing valued options (Haber and Knutson, 2010). The current connectivity findings may suggest that in social anxiety, there is a stronger need for ACC-based regulatory processes during incentive processing.

Reaction time data displayed a Group by Condition interaction for the general salience contrast. The social anxiety group had a higher reaction time on the neutral trials compared to reward and punishment trials, which could be suggestive of difficulty in processing ambiguous stimuli (Moscovitch and Hofmann, 2007; Moser et al., 2012). The subjective ratings also revealed trend-significant evidence for lower likeability ratings of the symbol indicating punishment than the one indicating reward in the social anxiety patients compared with controls. The higher likeability ratings in the control group for punishment compared to neutral trials may simply be indicative of a stronger motivation on those trials, since feedback is received on the performance. Taken together, the neural and behavioral findings provide some initial indication of a social-motivational imbalance in SAD, pointing at both valence-specific (striatal activation) and general salience effects (reaction time data and putamen-dACC connectivity). Hence, by explicitly testing motivational behavior and the anticipation of social incentives, the present findings extend the existing literature that was initially largely focused on fear processing and a disbalance in amygdala-centered fear circuitries (Etkin and Wager, 2007).

This study also extends findings from previous studies emphasizing the role of the striatum in behaviorally inhibited adolescents (Guyer et al., 2006) and adolescents with SAD (Guyer et al., 2012) when comparing monetary reward and punishment. Here we found valence-specific striatal effects when comparing social anxiety to controls. It is important to highlight the difference in incentive type (monetary in previous studies vs. social in our study), especially since it has been found that SAD is characterized by a differential effect; larger monetary reward, but smaller social reward activation (Richey et al., 2014). It is also relevant to acknowledge that the effects we observed are small, and independent replication is crucial. Ideally, future studies would include both social and non-social incentives, and reward and punishment trials. Another avenue for future research would be investigating whether the proposed motivational imbalance “shifts or normalizes” after a treatment which successfully increases reward sensitivity (e.g., Borgeat et al., 2009).

In line with predictions from a model of SAD development (Kimbrel, 2008) and empirical findings (Morgan et al., 2009; Kimbrel et al., 2010), SAD participants scored higher on the behavioral inhibition scale and lower on the behavioral activation scale. These findings, in combination with the putamen effects, may shed light on the relationship between individual differences and motivational valence-specificity. The controls showed a preference for reward anticipation (which fits the anticipatory affect model; Knutson and Greer, 2008) while this effect was unobserved in social anxiety. Striatal (dopaminergic) anticipatory activity might depend on the individual relevance (either implicitly or explicitly determined) of the upcoming reinforcer. Along this view, a recent fMRI study found support for a modulatory role of personal relevance (when valence is constant) in the ventral striatum (Carter et al., 2009). Another study found that harm avoidance scores correlated with ventral striatal activation during active avoidance of negative outcomes (Levita et al., 2012). However, the putamen-ACC connectivity findings showed a difference on general salience (valence-unspecific effect) between the controls and social anxiety group. The differences between social anxiety and controls in motivational processes may thus be complex, and dependent on the specific midbrain, striatal and frontal motivational subsystems. In general, the exact functional role of dopamine and striatal regions in the context of the anticipation of incentives is still under debate. Separate dopamine systems are thought to be related to either motivational salience, while others are specific to reward only (i.e., motivational value) (Matsumoto and Hikosaka, 2009; Bromberg-Martin et al., 2010). Other studies have found interactions between valence and action (go or no-go response) and specific regions in the motivational network (ventral tegmental area and ventral striatum; Guitart-Masip et al., 2011). Much work is needed to further elucidate the potential clinical relevance of motivational valence and salience effects.

## Limitations

There are a few other interpretational issues that need to be discussed. One point of concern is the specificity of the findings regarding the study population and incentives types. In this version of the SID task, we opted for a large number of the same trial types to optimize our main contrast of interest (reward > punishment anticipation). This came, however, at the expense of including another control condition (for example a non-social control condition) or applying a fully balanced design. Such a design would not only include congruent trials (happy faces signaling a fast response, angry faces a loss) as we have used here, but also incongruent trials (i.e., happy faces signaling a slow response, and angry faces signaling a fast response, as was used for example by Vrtièka et al. (2008). Future studies should apply these balanced designs to get a more specific view on valence differences in social incentive anticipation. In addition, a direct comparison with another anxiety patient group could increase the potential specificity of our findings. Moreover, in this study we used static faces that did not have direct personal relevance to the participants.

Several studies have used dynamic facial expressions (e.g., Trautmann et al., 2009) which have arguably more ecological validity. This same validity argument can be used for tasks designed to increase the personal relevance of stimuli. For example, in one study participants thought they would engage in a computer chat session with other peers, whose pictures were used as stimuli in the actual fMRI experiment (Guyer et al., 2008). Such adjustments could help increase the social nature of the task.

Lastly, most fMRI studies suffer from low statistical power, due the large amount of dependent variables (i.e., voxels) and often relatively small numbers of participants (Yarkoni, 2009; Button et al., 2013). Our current analytical approach partially addresses this concern by reducing the number of outcome variables to a single measure of neural activity related to motivational processing. However, we acknowledge that the sample size is small which, underscores the importance of independent replication of the current findings.

## Conclusions

Whereas controls show relatively heightened striatal response to cues signaling reward rather than avoiding punishment, SAD participants did not show this effect. These results imply that the relative motivational preference for social reward trials observed in controls is absent in SAD participants. In addition, altered connectivity between the putamen and the ACC in social anxiety may indicate stronger disruption of ACC control processes. These findings provide initial evidence for altered frontal-striatal involvement during social incentive anticipation in SAD, and highlight the importance for future research to focus on motivational processes in SAD.

### Conflict of interest statement

The authors declare that the research was conducted in the absence of any commercial or financial relationships that could be construed as a potential conflict of interest.
