# Supplementary material for: Neural sensitivity to social reward and punishment anticipation in social anxiety disorder
Source: Front Behav Neurosci. 2015 Jan 5;8:439. doi: 10.3389/fnbeh.2014.00439 (PMC4283602; doi:10.3389/fnbeh.2014.00439)

**Supplementary material**

“Neural sensitivity to social reward and punishment anticipation in Social Anxiety Disorder”

**S1. Voxel-wise analysis and results**

Group-level voxel-wise t-tests were performed on each of the subject level contrast maps, testing both main effects (across groups) and differences between groups. In order to control the voxel-wise false positive rate, we applied a cluster threshold of Z>2.3, and a corrected p<0.05, for effects within (a) an ROI mask related to reward and punishment processing and (b) the whole brain. The ROI mask was created using the meta-analytic database Neurosynth (Yarkoni et al., 2011) and consisted of the conjunction between the reverse inference statistical maps related to the terms “reward” and “punishment” (see supplementary figure 1). Grey matter density values were entered as covariates.

The vowel wise results showed two significant ROI corrected clusters for reward>baseline across groups and punishment>baseline across groups: the Putamen (x = -20, y =12 z = 4, k = 242) and thalamus (x = 4 y = -24 z = 6, k = 409). The general salience contrast (reward+punishment>baseline) showed two whole brain corrected clusters in the occipital cortex (x=-16, y=-88, z=-10, k=566 and x=6, y=-84, z=40, k=456). None of the other contrasts showed any significant effects, and none of the contrasts showed significant (whole brain or ROI corrected) between group differences.


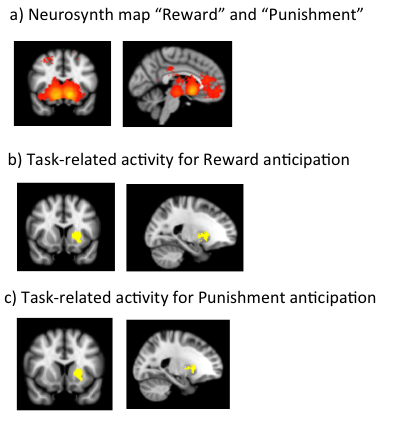


**Supplementary Figure 1.**

Statistical maps used in the analysis (a) meta-analytic statistical maps based on *Neurosynth* (Yarkoni et al., 2011) related to the terms “Reward” and “Punishment” which was then used as a ROI for (b) task related activity for reward >baseline, across subjects (c) punishment>baseline, across subjects. These later two maps where subsequently combined and beta values extracted, and used for further analysis.

**Supplementary Figure 2.**

Co-activation maps of left-putamen made using Neurosynth (Yarkoni et al., 2011).


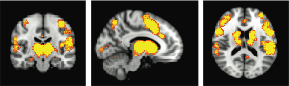

Supplement: Supplementary file 1 [file DataSheet1.DOCX]
